# Synthesis, Characterization, DFT, and In Silico Investigation of Two Newly Synthesized β-Diketone Derivatives as Potent COX-2 Inhibitors

**DOI:** 10.3390/bioengineering10121361

**Published:** 2023-11-27

**Authors:** Malahat Musrat Kurbanova, Abel Mammadali Maharramov, Arzu Zabit Sadigova, Fidan Zaur Gurbanova, Suraj Narayan Mali, Rashad Al-Salahi, Youness El Bakri, Chin-Hung Lai

**Affiliations:** 1 Organic Chemistry Department, Baku State University, Z. Khalilov 23, Baku 1148, Azerbaijan; amaharramov@bsu.edu.az (A.M.M.); arzu_sadigova@yahoo.com (A.Z.S.); 2 Department of Pharmacy and Biotechnology, Bioinformatics, University of Bologna, Via Marsala, 49/A, 40126 Bologna, Italy; fidangurbanova95@outlook.com; 3 Department of Pharmaceutical Sciences and Technology, Birla Institute of Technology, Mesra 835215, India; mali.suraj1695@gmail.com; 4 Department of Pharmaceutical Chemistry, College of Pharmacy, King Saud University, Riyadh 11451, Saudi Arabia; ralsalahi@ksu.edu.sa; 5 Department of Theoretical and Applied Chemistry, South Ural State University, Lenin Prospect 76, Chelyabinsk 454080, Russia; yns.elbakri@gmail.com; 6 Department of Medical Applied Chemistry, Chung Shan Medical University, Taichung 40241, Taiwan

**Keywords:** β-diketone, COX-2, DFT, Hirshfeld surface, ADME

## Abstract

Despite extensive genetic and biochemical characterization, the molecular genetic basis underlying the biosynthesis of β-diketones remains largely unexplored. β-Diketones and their complexes find broad applications as biologically active compounds. In this study, in silico molecular docking results revealed that two β-diketone derivatives, namely 2-(2-(4-fluorophenyl)hydrazono)-5,5-dimethylcyclohexane-1,3-dione and 5,5-dimethyl-2-(2-(2-(trifluoromethyl)phenyl)hydrazono)cyclohexane-1,3-dione, exhibit anti-COX-2 activities. However, recent docking results indicated that the relative anti-COX-2 activity of these two studied β-diketones was influenced by the employed docking programs. For improved design of COX-2 inhibitors from β-diketones, we conducted molecular dynamics simulations, density functional theory (DFT) calculations, Hirshfeld surface analysis, energy framework, and ADMET studies. The goal was to understand the interaction mechanisms and evaluate the inhibitory characteristics. The results indicate that 5,5-dimethyl-2-(2-(2-(trifluoromethyl)phenyl)hydrazono)cyclohexane-1,3-dione shows greater anti-COX-2 activity compared to 2-(2-(4-fluorophenyl)hydrazono)-5,5-dimethylcyclohexane-1,3-dione.

## 1. Introduction

Currently, the realm of β-diketones stands as a captivating focus of exploration owing to its noteworthy biological impact [1,2,3]. Analytical chemistry frequently employs β-diketones as a group of spectrophotometric reagents due to their remarkable ability to form complexes [4,5,6,7,8,9]. Additionally, derivatives of these compounds play a pivotal role in the treatment of inflammatory diseases with antioxidant and antiviral attributes [10,11,12]. Various β-diketones and their complexes serve as biologically active compounds [10,12]. In addition to substituted reagents and laser chelates [13], the chemical and photochemical catalysts [14] derived from these compounds are valuable in managing inflammatory diseases.

Cyclooxygenase-2, COX-2, facilitates the conversion of arachidonic acid into prostaglandin (PGH2). PGH2, a crucial precursor of prostacyclin, is expressed during inflammation. While COX-2 remains unexpressed under normal conditions in most cells, elevated levels are observed in cells where prostaglandins are upregulated during inflammation. Moreover, COX-2 expression is heightened in many cancers, possibly due to the conversion of its product, PGH2, into prostaglandin E2 by prostaglandin E2 synthase. This compound can stimulate cancer progression. Consequently, inhibiting COX-2 could offer benefits in preventing and treating cancer [15,16].

The effectiveness of nonsteroidal anti-inflammatory drugs (NSAIDs) lies in their inhibition of prostaglandin production by COX-1 and COX-2. NSAIDs that selectively inhibit COX-2 are less likely to induce gastrointestinal side effects compared to conventional drugs. However, they may contribute to cardiovascular events such as heart failure, myocardial infarction, and stroke. Numerous pieces of evidence suggest that this is linked to the suppression of COX-2-dependent cardioprotective prostaglandins, particularly prostacyclin [17]. The selective COX-2 inhibitors can selectively inhibit the COX-2 enzyme without inhibiting COX-1 due to the structural difference of COX-1 with respect to COX-2. For example, COX-1 contains isoleucine (Ile523) in its critical position, whereas COX-2 features valine (Val523) at the same site. Although such structural difference might be minor, it could result in substantial consequences, such as the binding packet of COX-2 being larger than that of COX-1, a secondary binding packet being unlocked, and the polar amino acid ARG513 being uncovered [18].

Moreover, except for aspirin (acetylsalicylic acid), which can irreversibly bind to the serine in the active center of cyclooxygenase through its acetyl group, thereby irreversibly inhibiting cyclooxygenase activity, the inhibitory effect of most NSAIDs on cyclooxygenase is reversible and therefore weak. Since this is the case, research on COX-2 inhibitors to replace NSAIDs has drawn attention [19]. This study presented the syntheses, characterizations, DFT studies, Hirshfeld surfaces, and energy frameworks of two new β-diketone derivatives. Moreover, an in silico investigation was performed into their capacities to behave as COX-2 inhibitors.

## 2. Materials and Methods

### 2.1. Chemistry

**Scheme 1:**
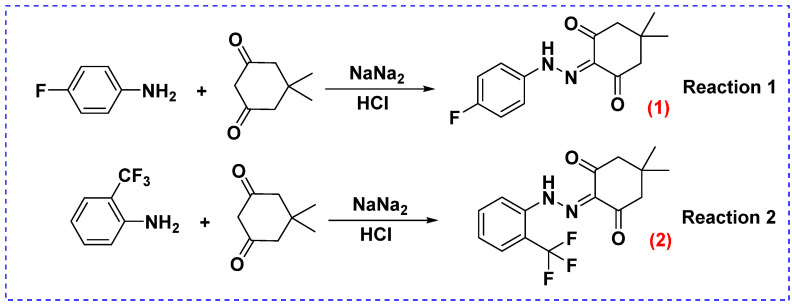
The synthesis of 2-(2-(4-fluorophenyl)hydrazono)-5,5-dimethylcyclohexane-1,3-dione (**1**) and 5,5-dimethyl-2-(2-(2-(trifluoromethyl)phenyl)hydrazono)cyclohexane-1,3-dione (**2**).

With this section, Scheme 1, Reaction 1 demonstrates the synthesis of target compound (**1**) [2-(2-(4-fluorophenyl)hydrazono)-5,5-dimethylcyclohexane-1,3-dione] by employing the diazotization of different aromatic amine by using 5,5-dimethylcyclohexane-1,3-dione. As a further output of the research, 5,5-dimethyl-2-(2-(2-(trifluoromethyl)phenyl)hydrazono)cyclohexane-1,3-dione (**2**) had been synthesized (Scheme 1, Reaction 2).

### 2.2. General

The response and purity of the substances were tracked using TLC (Sorbil). The examination of the substance structures was conducted employing the “Bruker APEX II CCD” diffractometer, operating at a temperature of 100 K, with λ_MoKα_-radiation, a graphite monochromator, and both ϕ- and ω-scanning capabilities, reaching a maximum 2θ value of 560. NMR spectra were recorded on Jeol 400 Mhz NMR spectrometer.

#### General Procedure for the Synthesis of 2-(2-(Aryl)hydrazono)-5,5-dimethylcyclohexane-1,3-diones (**1** and **2**)

**2-(2-(4-fluorophenyl)hydrazono)-5,5-dimethylcyclohexane-1,3-dione (1)**

In a three-necked flask, 6.25 mmol (0.69 g) of an aromatic amine and 6.25 mmol (0.35 g) of potassium hydroxide (KOH) were dissolved in distilled water. Subsequently, 0.0225 mol (1.55 g) of sodium nitrite (NaNO_2_) was dissolved in 2 mL of distilled water and introduced into the mixture, which was then subjected to stirring under the influence of a magnetic stirrer. To this mixture, 2 mL of hydrochloric acid (HCl) was added dropwise, maintaining a temperature of 0 °C, and the stirring continued for 30 min.

Following this, 6.25 mmol (0.88 g) of 5,5-dimethylcyclohexane-1,3-dione and 6.25 mmol (0.51 g) of sodium acetate (CH_3_COONa) were dissolved in 10 mL of ethyl alcohol (C_2_H_5_OH). The temperature of this new mixture was lowered to 0 °C, and it was added drop by drop to the previously prepared mixture, which was left to stir for an additional hour at 0 °C. The resulting product was then filtered and subjected to recrystallization in ethanol. (Yield: 74%), T_m.p_ = 198–200 °C, C_14_H_15_N_2_O_2_F; calculated for (%): C 64.12; H 5.72; N 10.68; F 7.25. Found (%): C 64.21; H 5.59; N 10.71, F 7.15. ^1^H-NMR (400 MHz) (DMSO-d6) δ: ppm; 0.97–1.09 (6H, 2CH_3_), 2.64–2.71 (4H, 2CH_2_), 6.61–6.99 (4H, CH-Ph), 8.21 (1H, NH). ^13^C-NMR (DMSO-d6) δ: m.h.; 26.92 (2CH_3_), 30.61 (C), 50.51, 51.12 (2CH_2_), 119.9, 116.58 (4CH, Ph), 137.91 (C=N), 137.9 (C-NH), 156.92 (C-F), 187.23 (2CO).

**5,5-dimethyl-2-(2-(2-(trifluoromethyl)phenyl)hydrazono)cyclohexane-1,3-dione (2)**

In a three-necked flask, 6.25 mmol (1.00 g) of an aromatic amine and 6.25 mmol (0.35 g) of potassium hydroxide (KOH) were dissolved in distilled water. Concurrently, 0.0225 mol (1.55 g) of sodium nitrite (NaNO_2_) was dissolved in 2 mL of distilled water and subsequently introduced into the mixture, followed by stirring under the influence of a magnetic stirrer. A solution of 2 mL of hydrochloric acid (HCl) was added dropwise to the mixture, and the stirring continued for a duration of 30 min under a temperature of 0 °C.

Subsequently, a solution comprising 6.25 mmol (0.88 g) of 5,5-dimethylcyclohexane-1,3-dione and 6.25 mmol (0.51 g) of sodium acetate (CH_3_COONa) in 10 mL of ethyl alcohol (C_2_H_5_OH) was prepared. The temperature of this solution was reduced to 0 °C, and it was gradually added drop by drop to the previously established mixture, which was left to stir for an additional hour under a temperature of 0 °C. The resultant product was subjected to filtration and subsequently underwent recrystallization in ethanol. (Yield: 73%), Tm.p. = 100–102 °C. C_15_H_15_N_2_O_2_F_3_; calculated for (%): C 57.69; H 4.80; N 8.97; F 18.26. Found (%): C 57.79; H 4.65; N 8.73; F 18.42. ^1^H-NMR (400 MHz) (DMSO-d6) δ: ppm; 0.97–1.06 (6H, 2CH_3_), 2.64–2.69 (4H, 2CH_2_), 6.55–7.49 (4H, CH-Ph), 8.16 (1H, NH). ^13^C-NMR (DMSO-d6) δ: m.h.; 26.71, 27.12 (2CH_3_), 29.94 (2C), 50.2, 51.6 (2CH_2_), 119.1, 133.2 (4CH, Ph), 135.49 (C-CF_3_), 137.34 (C-NH), 137.72 (C=N), 125.4 (CF_3_), 187.01 (2CO).

### 2.3. The DFT Optimizations

The gas-phase structure of the title compounds was optimized using the density functional theory. The DFT was calculated utilizing the B3LYP hybrid functional that is based on Becke’s idea of mixing the DFT exchange with the exact one (HF) using the B3 functional and combining the LYP correlation functional [20,21]. The B3LYP calculation was performed with the 6-311++G** basis set [22]. A similar theoretical level has been proved to obtain reliable results about the thiophene carboxamide derivatives [23]. Following the acquisition of the converged geometry, the harmonic vibrational frequencies were computed at the same computational level to verify that the imaginary frequency associated with the stationary point is zero. Both the optimization of the gas-phase geometry and the analysis of the vibrational frequencies were performed using the Gaussian 16 suite program [24].

### 2.4. The Hirshfeld Surface and Energy Framework Analysis

The delineation of a molecule within the condensed phase and the identification of distinct entities in molecular liquids and crystals represent fundamental concepts in chemistry. Leveraging Hirshfeld’s partitioning scheme, Spackman introduced a method for partitioning the electron distribution around a crystal into molecular fragments [25,26,27,28]. The proposed method could determine intermolecular interactions between specific molecules or within crystal structures. Since it has been raised from the Hirshfeld partition, the resulting surface is thus called the Hirshfeld surface. Lately, the energy framework has emerged as a potent tool for enhancing the clarity of understanding regarding how molecules arrange themselves in a crystal. In this review, both Hirshfeld surface analysis and the energy framework of the title compound were executed utilizing the CrystalExplorer program [29].

### 2.5. Molecular Docking

The structural and chemical features of the two studied β-diketones, which may inhibit COX-2, were discovered using theoretical computation in this contribution. The COX-2 enzyme structure was downloaded in the PDB format from the Protein Data Bank website (PDB code 4PH9). Since several references have already shown that the used molecular docking software might affect the accuracy of the result [30,31,32,33], three molecular docking tools, iGemDock, Autodock 4, Glide and Autodock vina, were then used [34,35,36,37,38]. The Autodock 4 and Autodock vina dockings were conducted with the aid of the AMDock program [39].

#### 2.5.1. The Preparations of the Ligands

The DFT-calculated results of the respective ligand was converted into the .mol file by the gOpenBabel [40]. 

#### 2.5.2. The Preparations of the Targets

**Figure 1:**
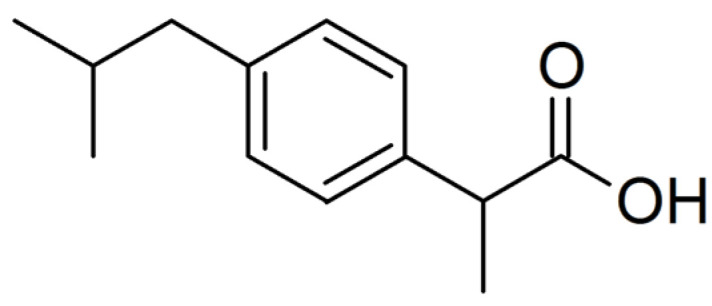
The structure of ibuprofen (**IBF**).

The .pdb format of the target was downloaded from the Protein Data Bank website and then treated by the UCSF Chimera 1.16 to delete the co-crystal ligands and water molecules [41,42]. Notably, one of the co-crustal ligands in the chosen COX-2 structure was ibuprofen (**IBF**) (see Figure 1). **IBF** is a NSAID commonly used to relieve pain, reduce fever, and reduce inflammation, and it was cited as the reference to investigate the inhibitory efficiencies of the studied β-diketones toward COX-2.

#### 2.5.3. Protocol Used for Docking Using Glide

We conducted molecular docking studies using Schrödinger Maestro Molecular Modeling software (Glide, Schrodinger, LLC, New York, NY, USA, 2023). The crystal structure of PREP (PDB: 4PH9) was chosen for its high resolution (1.81 Å) and the presence of co-crystallized ligand **IBF**, aiding binding site identification. Protein preparation involved the Maestro Protein Preparation Wizard (Maestro 13.2, Schrodinger, LLC, NY, 2023) with default preprocessing settings. Exceptions included missing loops and sidechains built using Prime, and “Epik” generated heteroatom states at pH 7.4. We eliminated acetate ions and glycerol, assigned hydrogen bonds, removed waters with <3 hydrogen bonds, and minimized the structure with the OPLS3e force field. Ligands, prepared with LigPrep using the OPLS-2005 force field, underwent no tautomer or stereoisomer generation. Docking utilized Glide with XP-peptide precision, employing receptor grids tailored for peptide docking with dimensions set at 15 Å (X and Y) and 10 Å (Z) around the ligand center. Ligands remained flexible, and the OPLS-2005 force field was applied throughout the process.

### 2.6. The ADMET Study 

ADME is an acronym for “Absorption, Distribution, Metabolism, and Elimination” in pharmacokinetics and pharmaceutics, which describes the distribution of drugs in an organism. ADME affects the levels of drugs and the exposure of drugs in the tissues, which affects the potency and bioactivity of compounds in drugs. In this study, the AMDE study was conducted in SwissADME [43].

### 2.7. Molecular Dynamics Simulation

In our molecular dynamics simulation (MDS) analysis, simulations were executed for the COX-2:1 and COX-2:2 complexes employing the “Desmond” module (Desmond, Schrödinger, Inc., New York, NY, USA, 2023, Free academic version) for a duration of 150 nanoseconds. The simulations utilized an explicit solvent model, incorporating TIP3P water molecules, and employed the OPLS-2005 force field. The simulation was performed within a periodic boundary solvation box with dimensions of 10 Å × 10 Å × 10 Å. To maintain a charge balance of 0.15 M, sodium ions (Na^+^) and NaCl solutions were introduced to mimic physiological conditions. This comprehensive approach followed the MD protocol from the previous studies [44,45,46,47,48,49].

## 3. Results

### 3.1. The Crystal Structures of the Studied Compounds

**Figure 2:**
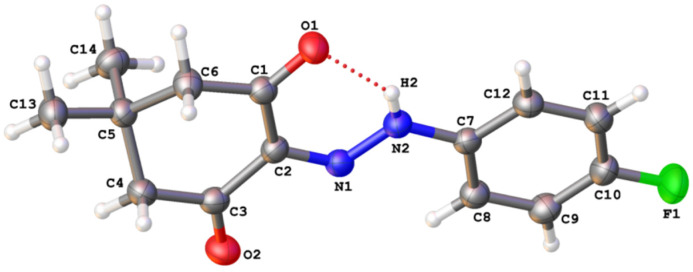
Molecular structure of 2-(2-(4-fluorophenyl)hydrazono)-5,5-dimethylcyclohexane-1,3-dione (**1**).

The triclinic structure of 2-(2-(4-fluorophenyl)hydrazono)-5,5-dimethylcyclohexane-1,3-dione (**1**) was deposited at the Cambridge Crystallographic Data Centre (CCDC 1475293) and depicted as shown in Figure 2. The lattice parameters of crystal a = 5.993(2) Å, b = 10.446(4) Å, c = 10.731(4) Å, α = 97.765(8)°, β = 102.860(8)°, γ = 98.925(8)°, space group P-1, Z = 2; V = 637.0(4) Å^3^, Dx = 1.368 g/cm^3^, μ = 0.102 mm^−1^. Crystal sizes 0.330 × 0.260 × 0.220 mm. The molecular structure of compound (**1**) is shown below. 

**Figure 3:**
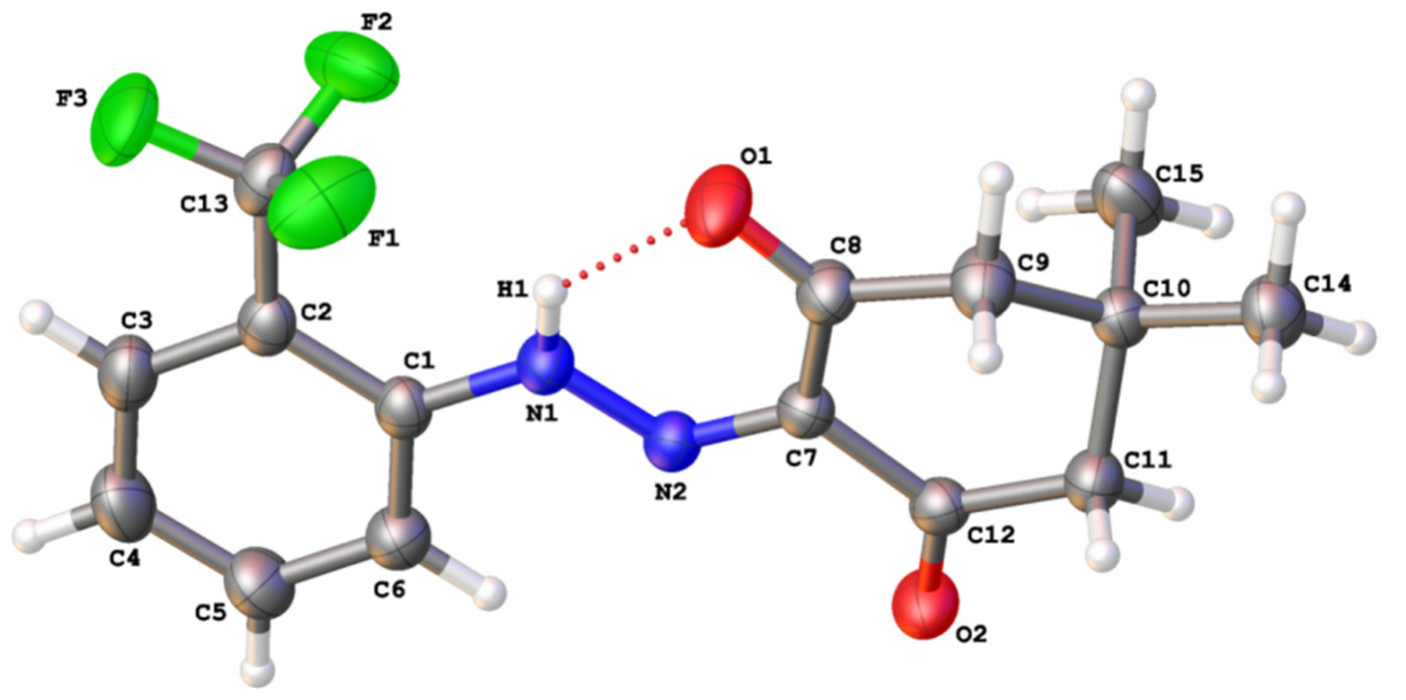
Molecular structure of 5,5-dimethyl-2-(2-(2-(trifluoromethyl)phenyl)hydrazono)cyclohexane-1,3-dione (**2**).

The monoclinic structure of 5,5-dimethyl-2-(2-(2-(trifluoromethyl)phenyl)hydrazono)cyclohexane-1,3-dione (**2**) was deposited at the Cambridge Crystallographic Data Centre (CCDC 1484656) and depicted as shown in Figure 3. The lattice parameters of crystal a = 15.5610(12) Å, b = 6.1069(5) Å, c = 15.6267(12) Å, β = 97.3588(13)°, V = 1472.8(2) Å^3^, Z = 4, space group P21/n, Dx = 1.315 g/cm^3^, μ = 0.120 mm^−1^. Crystal sizes 0.630 × 0.220 × 0.150 mm.

### 3.2. The DFT-B3LYP Study

**Figure 4:**
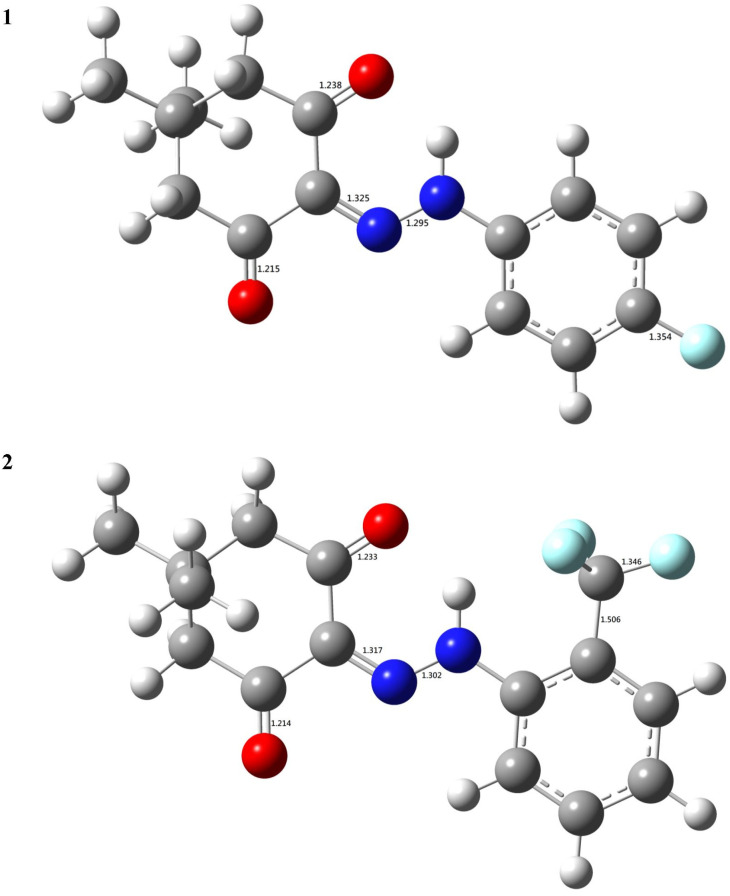
The B3LYP-optimized geometries of the title compounds (the bond lengths in Å).

In order to elucidate the connection between intrinsic electronic properties and the chemical reactivities (biological activities) of the title compounds, a gas-phase density functional theory (DFT) functional study was conducted using B3LYP. The B3LYP-optimized geometries are depicted in Figure 4 and Supplementary Materials for reference. As depicted in Figure 4, the individual bond length is as expected, which shows that the chosen theoretical level should be reliable. Moreover, the two C=O groups in both **1** and **2** have a difference in the bond length. The difference in the two C=O bond lengths was 0.023 Å, and 0.019 Å for **1**, and **2**, respectively. The converged geometry of the title compound, which was represented by the Cartesian coordinates of the respective atoms, was provided as the supporting information.

**Figure 5:**
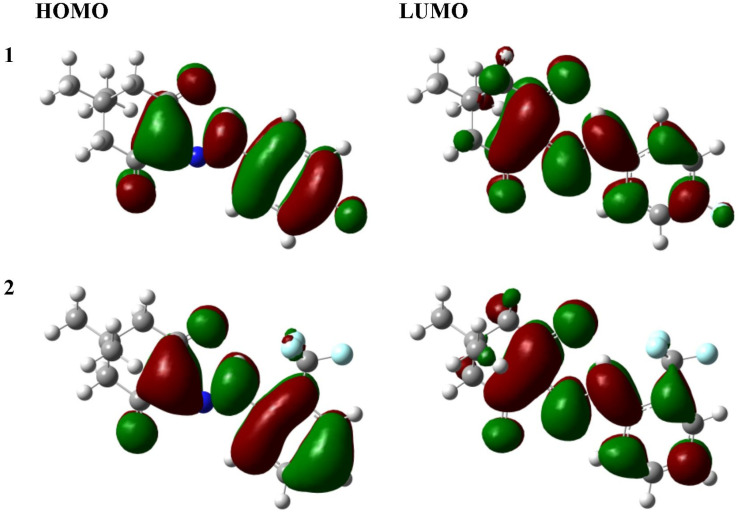
The frontier orbital of the title compounds (the isovalue = 0.02 a.u.).

The pivotal aspect of the frontier orbital theory lies in its emphasis on the highest occupied and lowest unoccupied molecular orbitals (HOMO and LUMO). Instead of solely contemplating the total electron density within the nucleophile, it is more instructive to focus on the electron density distribution within a molecule’s HOMO. Electrons in the HOMO exhibit the highest probability of engaging in nucleophilic attacks. Conversely, the electron density distribution in the LUMO serves as a reliable indicator for characterizing electrophilicity. Therefore, the frontier orbitals of the title compounds were further investigated in this study. As depicted in Figure 5, the frontier orbital of **1** or **2** was the linear combination of the π or π* orbital of the π-bonded moieties within **1** or **2**.

**Figure 6:**
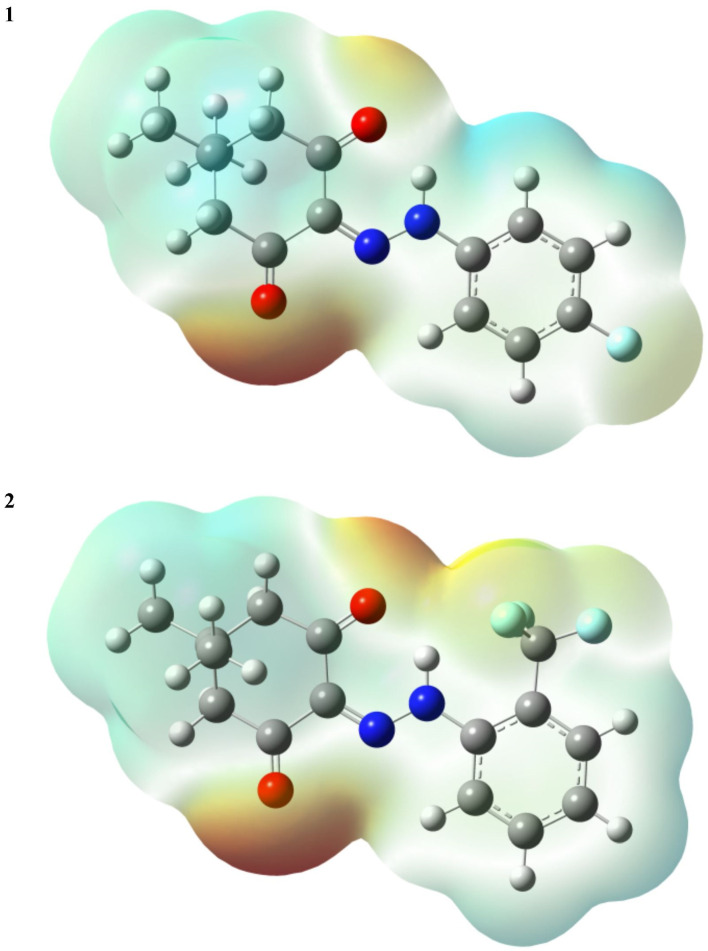
The MEPs of the title compounds (the isovalue = 0.0004 a.u.).

Molecular electrostatic potentials (MEPs) serve as a fundamental metric for gauging the strength of interactions among nearby charges, nuclei, and electrons at specific positions. This enables the examination of charge distribution and associated molecular properties. To enhance the interpretability of electrostatic potential information, a visual representation utilizing different colors was employed. Generally, regions in red signify the lowest electrostatic potential, rendering them susceptible to electrophilic attacks. Conversely, regions in blue indicate the highest electrostatic potential, making them susceptible to nucleophilic attacks. In this investigation, the total density matrix was utilized to derive the total density of the title compounds, and the resultant MEP was mapped onto their surfaces. As illustrated in Figure 6, oxygen atoms in the title compounds were identified as the sites prone to nucleophilic attack. Despite fluorine atoms possessing higher electronegativity than oxygen, those in both compounds **1** and **2** did not reside in the reddest region, where the MEP is not particularly negative. This can be elucidated by acknowledging that contributions to the molecules’ MEP arise from both electrons and nuclei.

### 3.3. Hirshfeld Surface Analysis

**Figure 7:**
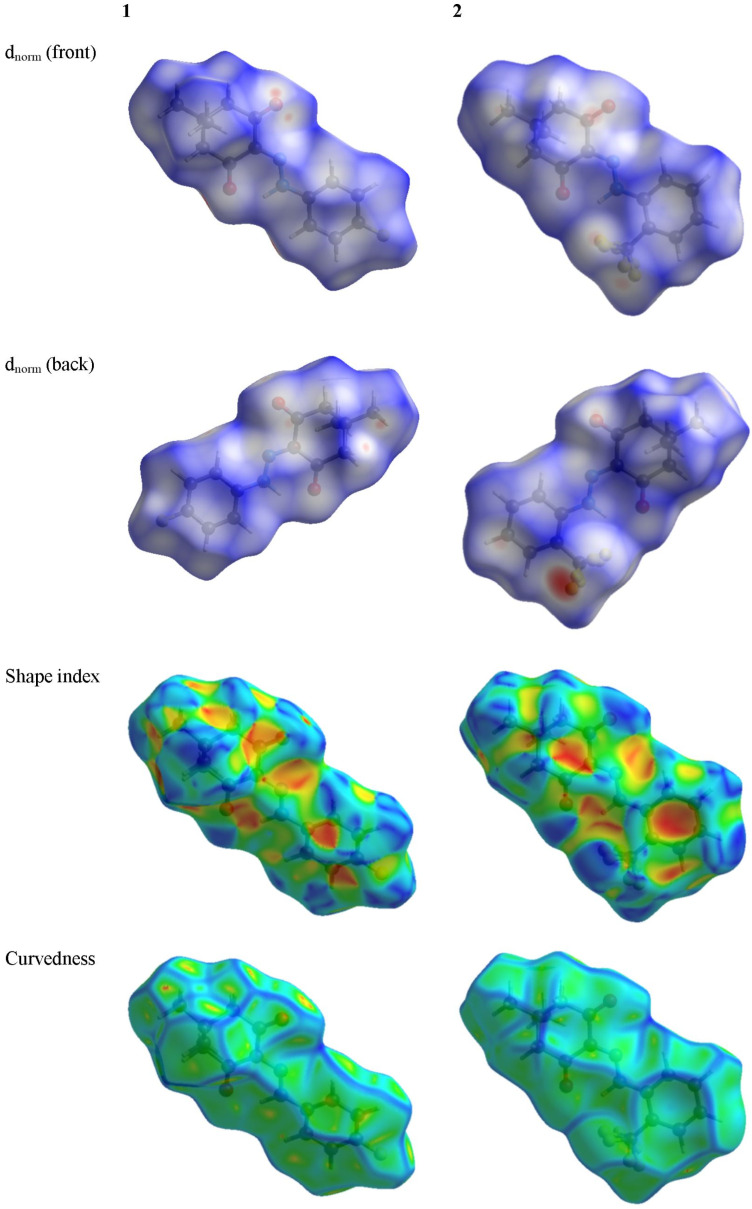
The Hirshfeld surface of the title compounds.

The Hirshfeld surfaces of the title compounds at standard resolution are illustrated in Figure 7. Transparency was applied to these surfaces to enable the visualization of the molecular moieties in a consistent orientation across all structures for which they were computed. The 3D d_norm_ surfaces serve to identify closely positioned intermolecular interactions. Negative (positive) values indicate that the intermolecular contacts are shorter (longer) than the van der Waals radii. The d_norm_ values are color-coded in red, white, or blue. Red regions signify closer contacts with negative d_norm_ values, whereas blue regions indicate longer contacts with positive d_norm_ values. Additionally, white areas represent contacts at the van der Waals separation with a zero value.

Comparing the shape index and curvedness of **1** with those of **2**, we can find that the π-π stacking interaction should be stronger in crystalline **2** than crystalline **1**. 

**Figure 8:**
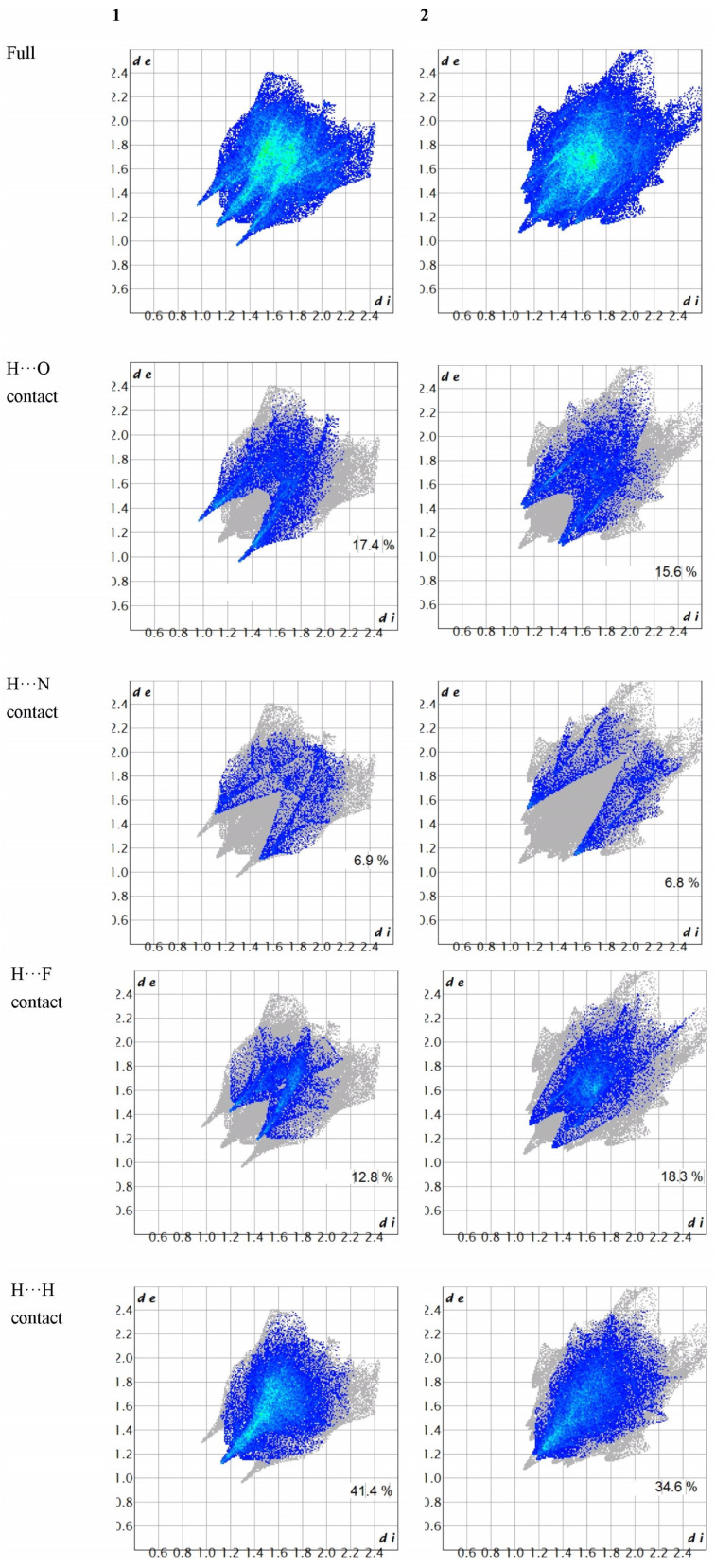
The 2D fingerprint plots of the title compounds.

The 2D fingerprint plots elucidate the interaction between the specific atom pairs and facilitate the dissection of the overall fingerprint into contributions from various interaction types. Employing a standard view ranging from 0.6 to 2.4, with the de and di distance scales depicted on the graph axes and incorporating reciprocal contacts (Figure 8), it was observed that the most noteworthy interaction involving hydrogen in both title compounds was the H···H contact. A comparison between the 2D fingerprint plots of **1** and those of **2** revealed that the H···F contact exhibited a more substantial contribution to the intra- or intermolecular interactions in **2** compared to those in **1**.

### 3.4. Energy Framework Analysis

**Figure 9:**
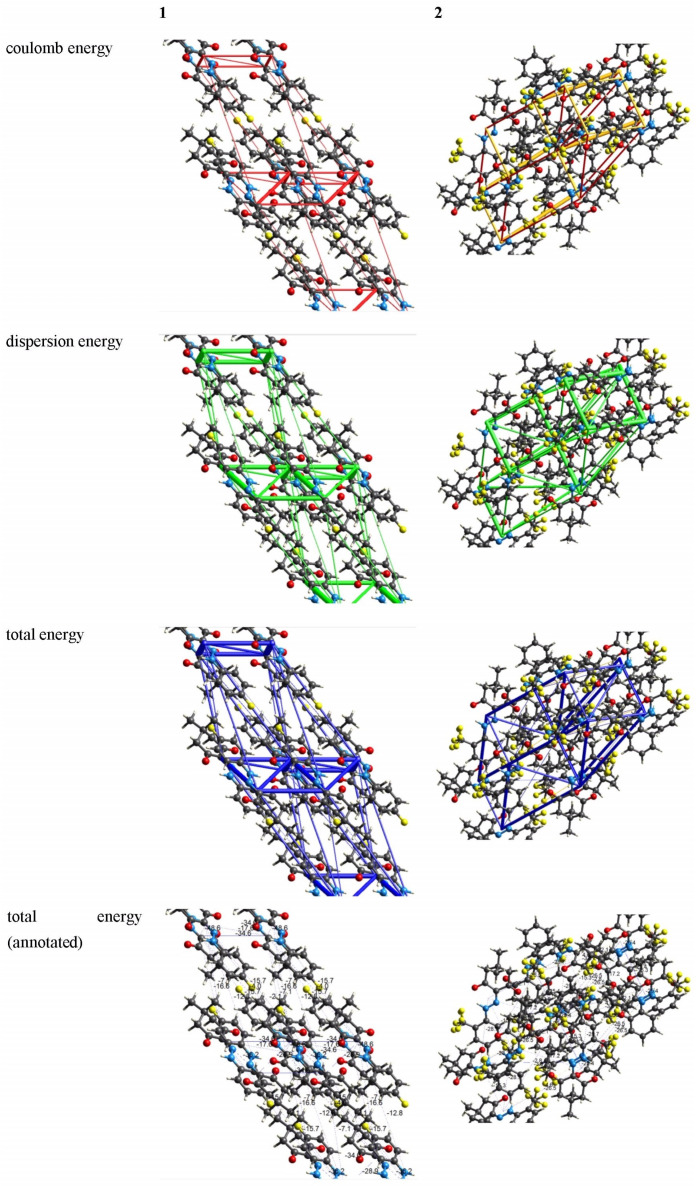
The energy framework of the title compounds (tube size: 60, and cutoff: 5.00 kJ/mol).

Energy frameworks were constructed based on interaction types (electrostatic, polarization, dispersion, and exchange-repulsion) utilizing density functional theory (CE-B3LYP) in conjunction with the 6-31G(d,p) basis set. A cluster of molecules within a 3.8 Å radius was formed and considered around the central molecule, after which energy calculations were conducted. The interaction energy can be partitioned into

(1) $$\begin{array}{c} E\_\mathrm{tot}=k\_\mathrm{eleE}\_\mathrm{ele}+k\_\mathrm{polE}\_\mathrm{pol}+k\_\mathrm{disE}\_\mathrm{dis}+k\_\mathrm{repE}\_\mathrm{rep} \end{array}$$

where k represents the scale factor, E_ele = electrostatic component, E_pol = polarization energy, E_dis = dispersion energy and E_rep = exchange-repulsion energy. As indicated in Table 1 and Table 2, the results demonstrate that the dispersion interaction significantly influences the interaction between the central molecule and its adjacent molecules in both crystalline **1** and **2**. It is noteworthy that, despite the scale factor of the electrostatic component (k_elec) being the largest among all scale factors for both **1** and **2** (as detailed in Table 3), the dispersion interaction plays a predominant role. These interactions are visually depicted in Figure 9.

### 3.5. The Molecular Docking Study

As mentioned earlier, β-diketones have been widely used as biologically active compounds, and then chosen to study and emphasize their possible target. According to the predicted result of the SwissTargetPrediction [50], the most probable target for **1** is COX-2. Hence, the anti-COX-2 activities of **1** and **2** were studied by the molecular docking in this study. **IBF** was taken as the reference to investigate the anti-COX-2 activities of the titled chemicals. Therefore, the .cif file of the **IBF** was downloaded from the PDB and converted into a .mol2 file by the gOpenBabel program [40]. Then, the MM2 method implemented in the Chem3D program was used to minimize the structure of **IBF**. Afterward, the geometry-optimization result of **IBF** was also saved as a .mol2 file. COX-2 (Cyclooxygenase-2) is a crucial enzyme involved in pain medication. When tissues are injured or inflamed, COX-2 is upregulated. It catalyzes the conversion of arachidonic acid into prostaglandins, particularly PGE2. Prostaglandins are lipid molecules that act as local messengers, sensitizing nerve endings to pain signals and promoting inflammation. PGE2 lowers the pain threshold by stimulating pain receptors (nociceptors) and increasing blood flow to the injured area. This amplifies pain perception and triggers the inflammatory response. Blocking COX-2 with specific inhibitors like NSAIDs (nonsteroidal anti-inflammatory drugs) reduces prostaglandin production, ultimately alleviating pain and inflammation.

**Figure 10:**
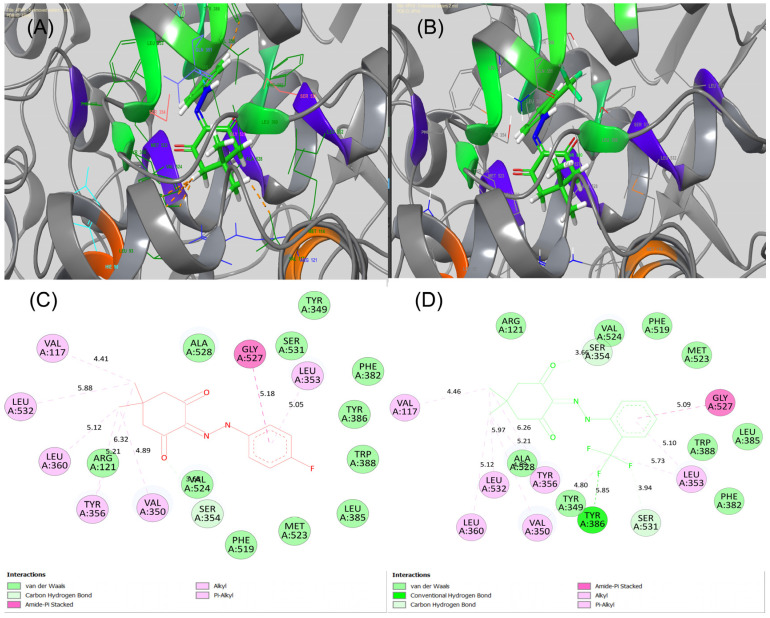
(**A**,**B**) 3D-binding poses and (**C**,**D**) 2D-binding poses for Compounds **1** and **2** against the active site pocket of COX-2 (PDB code 4PH9), respectively. This figure was created by using Discovery Studio Visualizer, 2023.

Thus, for the synthesized derivatives, we aimed to analyse the possibility of bindings of compounds **1** and **2** toward COX-2 (PDB ID:4PH9; Resolution: 1.81 Å; R-Value Observed: 0.162). Before starting the actual docking, we validated the entire protocol with Glide, Schrodinger, LLC, NY, 2023 and the concern value of RMSD was obtained as 0.50 Å for re-docking the co-crystallized ligand, **IBF**. As depicted in Figure 10C, compound **1** had the majority of the hydrophobic interactions with amino acid residues such as Val117, Leu532, Leu360, Tyr356, Val350, Leu353, and Val524. It is also worth mentioning that we noticed one amide-Pi stacking with amino acid residue Gly527. Conversely, compound **2** had hydrophobic interactions with Val117, Leu532, Leu360, Leu353, and Tyr356 (Figure 10D). Moreover, the fluorine atom had interacted with Tyr386 via H-bonding. The amide-Pi interacting residue, i.e., Gly527 was retained for compound **2** as similar to **1**. It is noticeable that the appearance of H-bond for compound **2** led the enhanced binding of COX-2, as evident from docking scores (Glide Dock Score, XP: –9.36 and –9.79 kcal/mol, respectively).

**Figure 11:**
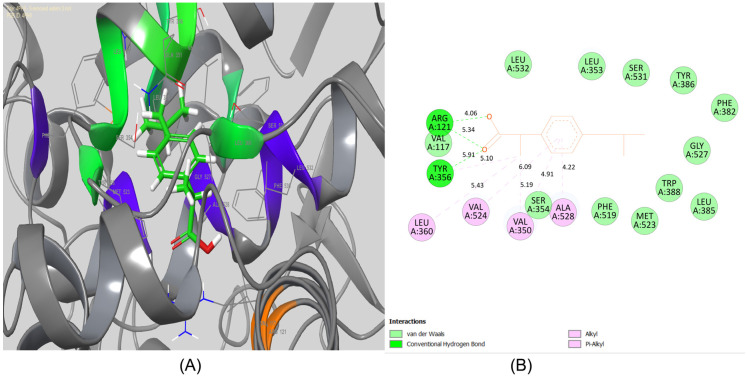
(**A**) 3D binding pose and (**B**) 2D-binding pose for compound **IBF** against the active site pocket of COX-2 (PDB code 4PH9). This figure was created by using Discovery Studio Visualizer, 2023.

The standard **IBF**, however, showed interactions with amino acid residues like Arg121, Val117, and Tyr 356 via H-bonding (Figure 11) (Glide Dock Score, XP: -7.98 kcal/mol). Other hydrophobic interactions were also seen for Leu360, val524, Val350, and Ala528 (alkyl or π-π interactions). 

Notably, the Autodock 4 and Autodock vina were both performed via AMDock program [39], in which the pH was set to be 7.4 and the other docking parameters were set automatically by the used program. Table 4 listed the comparison of the anti-COX-2 activities of the titled compounds and **IBF** obtaining by the iGemDock program [34,35]. Table 4 shows that both **1** and **2** have better anti-COX-2 activity than the chosen reference. Furthermore, the activity of **1** was larger than that of **2**, which may be due to the fact **1** could form stronger hydrogen bonding with COX-2 than **2** could. Both of the Autodock 4 and Autodock vina docking results were summarized as Table 5 showed. Similar to the iGemDock results, both the Autodock 4 and Autodock vina results also have shown that **1** and **2** have larger anti-COX-2 activity than the reference, **IBF**. However, the Autodock vina results showed that **2** should have a larger activity than **1**, which disagreed with the iGemDock results.

### 3.6. Molecular Dynamics Analysis

**Figure 12:**
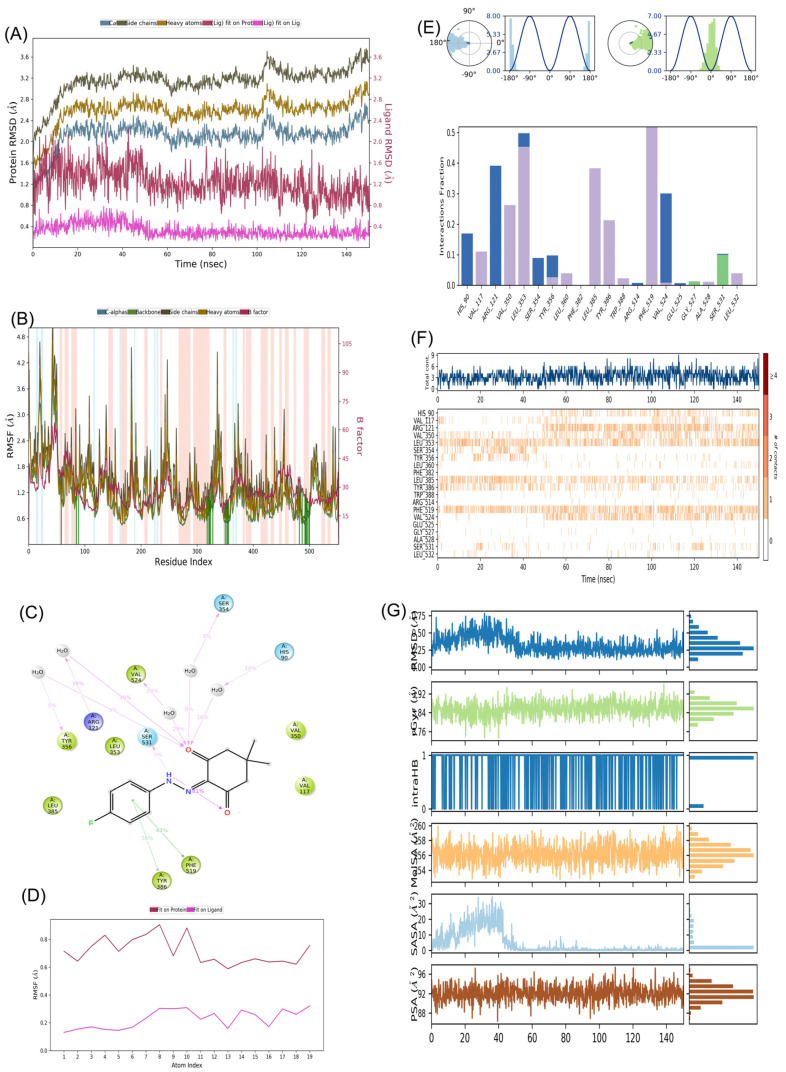
MD simulation analysis for complex COX-2 (PDB code 4PH9):1 (**A**) RMSD plot; (**B**) RMSF analysis; (**C**) Ligand–Protein contact plot; (**D**) Ligand RMSF; (**E**) Ligand torsion profile; (**F**) Protein–Ligand interaction plot; (**G**) a timeline representation plot representing such interactions with amino acid residues over the simulation period of 150 ns for compound **1**.

**Figure 13:**
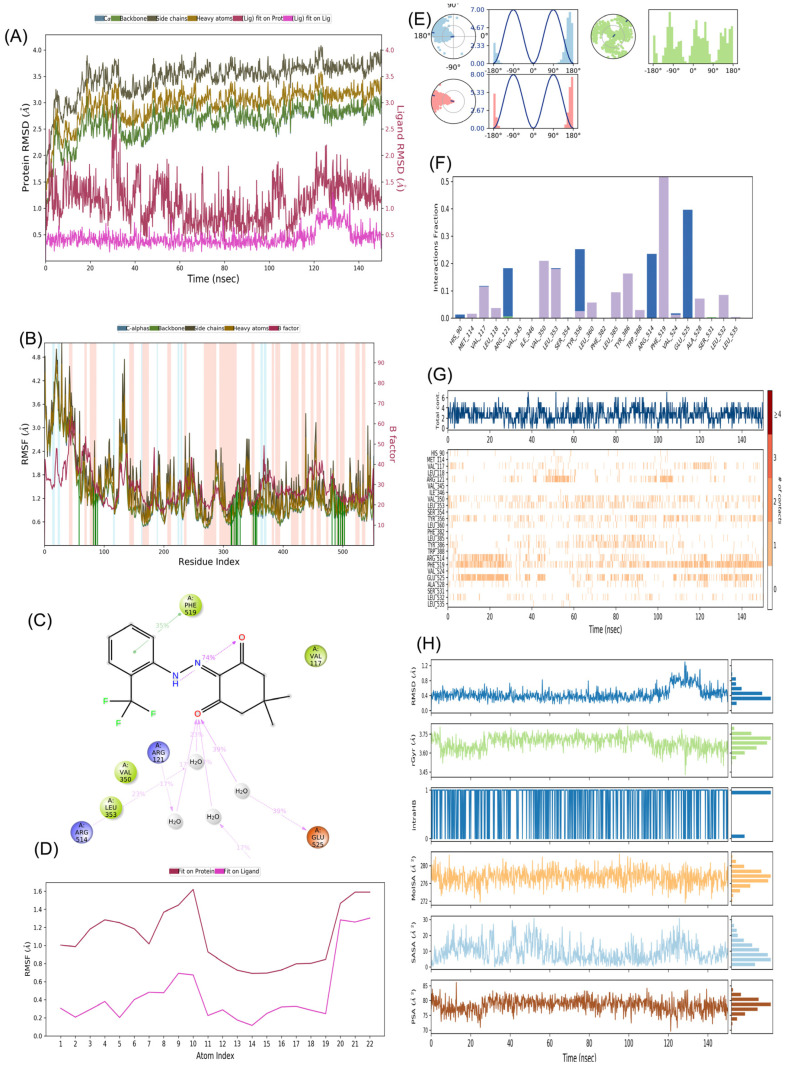
MD simulation analysis for complex COX-2 (PDB code 4PH9):2 (**A**) RMSD plot; (**B**) RMSF analysis; (**C**) Ligand–Protein contact plot; (**D**) Ligand RMSF; (**E**) Ligand torsion profile; (**F**) Protein–Ligand interaction plot; (**G**) a timeline representation plot representing such interactions with amino acid residues over the simulation period of 150 ns and (**H**) Ligand properties for compound **2**.

We assessed the stability of both-docked ligands, denoted as COX-2:1 and COX-2:2, using a 150 ns molecular dynamics simulation (MD) carried out with the “Desmond” software by Schrodinger, LLC in 2023 (refer to Figure 12 and Figure 13). The MD system comprised a combined total of 58,768 atoms for COX-2:1 and 57,055 atoms for COX-2:2, inclusive of 16,624 and 16,022 water molecules, respectively. In the case of the COX-2:1 complex, 553 residues with 8859 atoms were engaged, while for COX-2:2, the involvement comprised 553 residues with 8858 atoms. In our dynamic simulations, we gauged the average atomic displacements over a specific time frame, employing the “RMSD” (Root Mean Square Deviation) parameter. Our RMSD analysis consistently revealed stable conformations. When ligands **1** and **2** were bound to the target 4PH9, the C_α_-RMSD backbone values remained below 2.8 Å and 2.0 Å, respectively (refer to Figure 12A,B and Figure 13A,B). Additionally, the “Lig_fit_Prot” values consistently stayed below 2.5 Å for both simulations (see Figure 12B and Figure 13B), affirming the stability of the entire complexes over the 0–150 ns duration. We delved into local protein chain variations using the “RMSF” (Root Mean Square Fluctuation) plot (Figure 12B and Figure 13B), showing minor fluctuations in selected proteins without significant changes, highlighting the expected flexibility of these residues. Notably, we observed fewer fluctuations in amino acid residues for COX-2:2 compared to COX-2:1. Examining the “protein–ligand” interaction plot (Figure 12C), we discovered significant interactions, including hydrophobic interactions with amino acid residues Val117, Val350, Leu353, Tyr356, Leu360, Leu385, Tyr386, Phe519, and Leu532, but no ionic interactions (Figure 12F) in the complex COX-2:1. Water bridges formed with His90, Arg121, Leu353, Ser354, and Val524 for compound **1**. Amino acid residues Gly527 and Ser531 established H-bonds. Similarly, the “protein–ligand” interaction plot (Figure 13F) for compound **2** displayed significant interactions, including hydrophobic interactions with amino acid residues Val117, Val350, Leu353, Tyr356, Leu360, Leu385, Tyr386, Phe519, and Leu532, with no ionic interactions. Water bridges were observed with His90, Arg121, Leu353, Ser354, and Val524. 

Figure 12G and Figure 13G presents a timeline representation of these interactions with amino acid residues over the 150 ns simulation period.

Figure 12C presents a “ligand–protein” contact plot, highlighting interactions that persisted for over 5.0% of the simulation time within the selected trajectory (0.00–150.00 ns) for compound **1**. Notable amino acid residues included Tyr356 (5% H-bonding), Arg121 (39% H-bond), Ser531 (5% H-bond), Phe519 (43% pi–pi interactions), and Tyr386 (16% pi–pi interactions). Similarly, Figure 13C illustrates a “ligand–protein” contact plot for compound **2**, revealing interactions lasting more than 5.0% of the simulation time in the same trajectory. Compound **2** had interacted with two key amino acids, as Glu525 and Tyr356 via H-bonding. Furthermore, the “ligand torsions plot” (Figure 12E and Figure 13E) succinctly summarizes conformational changes in each rotatable bond (RB) within the ligand over the entire simulation duration (0.00–150.00 ns) for **1** and **2**, respectively. In conclusion, our results substantiate the stable conformations of the ligand–protein complexes over the 150 ns simulation period. The ligand RMSF plot for **2** (Figure 13D) had more fit to the protein site compared to 1 (Figure 12D), suggesting better binding of **2** toward COX-2.

### 3.7. The ADMET Study

**Figure 14:**
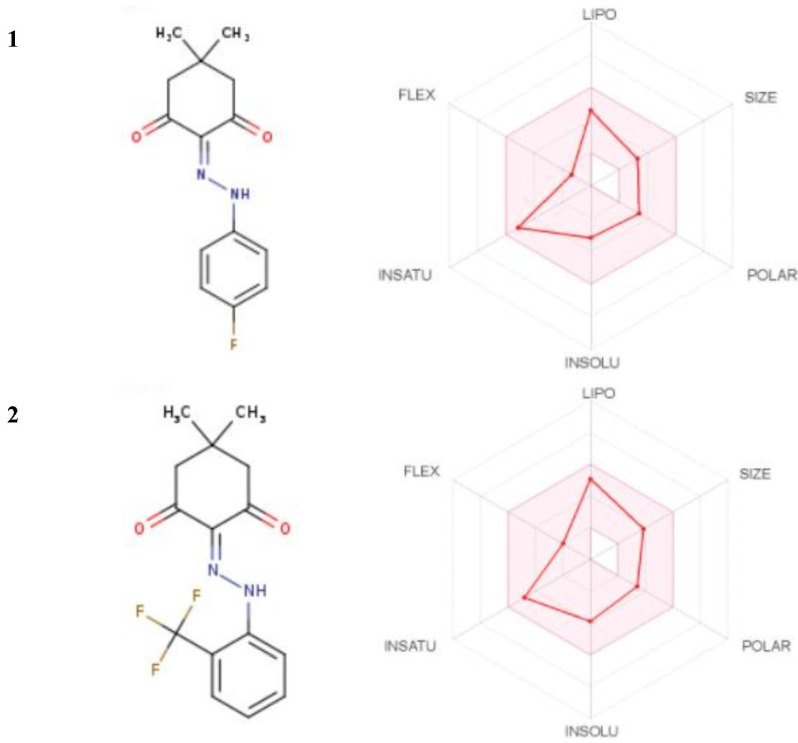
The SwissADME plots of drug-likeness of the title compounds. The pink area represents the optimal range for each property (lipophilicity: XLOGP3 between −0.7 and +5.0, size: MW between 150 and 500 g/mol, polarity: TPSA between 20 and 130 Å^2^, solubility: logS not higher than 6, saturation: fraction of carbons in the sp^3^ hybridization not less than 0.25, and flexibility: no more than 9 rotatable bonds).

The drug-likeness including the Lipinski’s parameters and pharmacokinetic properties of the titled compounds [51] were investigated by using the SwissADME online server. After analyzing 90% of orally active drugs, Lipinski reported that the drugs that reached phase II clinical testing have molecular weights ≤ 500 g/mol, number of hydrogen-bond donors (no. of H-bond donors) ≤ 5, and number of hydrogen-bond acceptors (no. of H-bond acceptors) ≤ 10 [51]. The SwissADME plot predicts additional physicochemical parameters associated with drug-likeness such as lipophilicity, topological polar surface area, solubility, and saturation [52,53,54,55,56,57,58,59,60,61]. The results could be summarized as shown in Figure 14 and Table 6. The SwissADME plot of the drug-likeness of the title compounds showed that all the physicochemical properties of the compounds were within the desirable range. The predicted pharmacokinetics showed that the skin permeation (logKp) was −6.08 cm/s, and −5.83 cm/s for **1**, and **2**, respectively. The more negative logKp of **1** indicated that **1** was less skin permeant than **2** [61].

## 4. Conclusions

In this study, two following β-diketone derivatives 2-(2-(4-fluorophenyl)hydrazono)-5,5-dimethylcyclohexane-1,3-dione, and 5,5-dimethyl-2-(2-(2-(trifluoromethyl)phenyl)hydrazono)cyclohexane-1,3-dione were synthesized and characterized spectroscopically. Since β-diketones have been widely used as biologically active compounds, we studied them through molecular docking, molecular dynamics and the DFT as powerful techniques to investigate and evaluate the biological activity of our compounds against COX-2. Then, we analyzed ADMET further by the SwissADME plots and discovered that our compounds are well adsorbed and neither toxic nor carcinogenic. Our structures were also optimized through Hirschfeld surface analysis and energy framework, using the Crystal Explorer program. According to the results of the molecular docking studies, the title chemicals might have larger anti-COX-2 activities than **IBF**. However, since the predicted relative activity results of Glide, iGemdock, and Autodock vina were opposite, we were convinced that the in vitro anti-COX-2 activities of β-diketone derivatives are worth further study. To have more clarity, the MD study pointed out that compound **2** had better binding toward COX-2 and also obtained the best docked candidate from the Glide calculations. Furthermore, we firmly believe that the results of this paper should enlighten a broad spectrum in the research field of developing NSAIDs as COX-2 inhibitors.

## Figures and Tables

**Table 1:** The energy framework of **1**. (energy in kJ/mol and R in Å).

N	Symop	R	Electron Density	E_ele	E_pol	E_dis	E_rep	E_tot
2	x, y, z	10.45	B3LYP/6-31G(d,p)	−0.2	−0.1	−10.1	3.4	−7.1
1	−x, −y, −z	13.23	B3LYP/6-31G(d,p)	−6	−0.4	−7	0	−12.8
2	x, y, z	5.99	B3LYP/6-31G(d,p)	−12.4	−3.8	−37.8	23	−34.6
1	−x, −y, −z	5.48	B3LYP/6-31G(d,p)	−19.8	−6.3	−51.1	34.8	−48.6
1	−x, −y, −z	6.89	B3LYP/6-31G(d,p)	−6.5	−5.5	−14.7	9.8	−17.6
1	−x, −y, −z	6.55	B3LYP/6-31G(d,p)	−22.8	−5.5	−24.3	33.2	−28.9
1	−x, −y, −z	5.63	B3LYP/6-31G(d,p)	−5.7	−1.2	−49.7	22.7	−36.2
2	x, y, z	12.82	B3LYP/6-31G(d,p)	−5.5	−0.4	−11	0	−15.7
1	−x, −y, −z	12.43	B3LYP/6-31G(d,p)	−2.5	−0.8	−15.4	0	−16.6
1	−x, −y, −z	15.97	B3LYP/6-31G(d,p)	−1	−0.1	−1.2	0	−2.1
1	−x, −y, −z	15.11	B3LYP/6-31G(d,p)	−0.3	0	−4.2	0	−4

**Table 2:** The energy framework of **2**. (energy in kJ/mol and R in Å).

N	Symop	R	Electron Density	E_ele	E_pol	E_dis	E_rep	E_tot
0	x + 1/2, −y + 1/2, z + 1/2	12.67	B3LYP/6-31G(d,p)	0	−5.8	0	0	−4.3
0	−x + 1/2, y + 1/2, −z + 1/2	7.88	B3LYP/6-31G(d,p)	−2.4	−6.8	−27.9	8	−26.8
0	x, y, z	6.11	B3LYP/6-31G(d,p)	22.8	−18.8	−44.2	16.7	−18
0	−x, −y, −z	8.12	B3LYP/6-31G(d,p)	15.3	−24.3	−49	27.4	−27.5
0	X + 1/2, −y + 1/2, z + 1/2	11.78	B3LYP/6-31G(d,p)	−14.2	−5.1	−13.5	4.4	−27.9
0	−x, −y, −z	7.94	B3LYP/6-31G(d,p)	35.1	−17.5	−23.3	1.4	4.8
0	−x + 1/2, y + 1/2, −z + 1/2	8.84	B3LYP/6-31G(d,p)	−15.4	−4.1	−16.4	5.5	−30.2
0	−x, −y, −z	9.03	B3LYP/6-31G(d,p)	3.9	−17.7	−17.2	7.2	−19.5
0	−x, −y, −z	8.87	B3LYP/6-31G(d,p)	−4.2	−5.6	−21.5	30.2	−8.7

**Table 3:** The scale factor for CE-B3YLP model energies with B3LYP/6-31G(d,p) monomer electron densities.

	k_ele	k_pol	k_disp	k_rep
1	1.057	0.651	0.901	0.811
2	1.057	0.740	0.871	0.618

**Table 4:** The iGemDock docking results.

	1	2	IBF
Energy	−86.7525	−85.0493	−81.2119
Van Der Waals	−70.4011	−75.6551	−81.2119
Hydrogen Bond	−16.3514	−9.39419	0
Electrostatic	0	0	0

**Table 5:** The docking results using the AMDock program.

	1		2		IBF	
	Autodock 4	Autodock Vina	Autodock 4	Autodock Vina	Autodock 4	Autodock Vina
Affinity (kcal/mol)	−8.57	−8.4	−8.4	−8.9	−7.7	−6.7
Estimated K_i_	522.58	696.25	696.25	299.41	2.27	12.27
K_i_ units	nM	nM	nM	nM	uM	uM
Ligand Efficiency	−0.45	−0.44	−0.38	−0.40	−0.51	−0.45

**Table 6:** Summary of SwissADME predicted physicochemical descriptors and ADMET parameter of the titled compounds.

	1	2
Physicochemical Properties		
Molecular weight in g/mol (≤500)	262.28	312.29
Saturation: fraction of carbons in the sp^3^ hybridization (not less than 0.25)	0.36	0.40
Lipophilicity: XLOGP3 (desirable between −0.7 and +5.0)	2.56	3.34
No. rotatable bonds (not more than 9 rotatable bonds)	2	3
No. H-bond acceptors (H-bond acceptor ≤ 10)	4	6
No. H-bond donors (H-bond donors ≤ 5)	1	1
Topological polar surface area TPSA (between 20 and 130 Å^2^)	58.53	58.53
Solubility		
log S (Ali)	−3.44	−4.25
log S (ESOL)	−3.18	−3.88
Pharmacokinetic properties		
GI absorption	High	High
P-glycoprotein substrate	No	No
Skin permeation (logK_P_ in cm/s)	−6.08	−5.83
BBB permeation	Yes	Yes
Cytochromes P450 1A2, 2C19, 2C9, 2D6. 3A4 inhibitor	Only for 1A2	Only for 2C19
Bioavailability score	0.55	0.55

## Data Availability

Data available on request due to restrictions e.g., privacy or ethical.

## Supplementary Materials

The following are available online at https://www.mdpi.com/article/10.3390/bioengineering10121361/s1, DFT-converged geometries, checkCIF files of the titled chemicals, and the whole amino acid sequences of the COX-1 and COX-2 enzymes.
