# Supplementary material for: Synthesis, Characterization, DFT, and In Silico Investigation of Two Newly Synthesized β-Diketone Derivatives as Potent COX-2 Inhibitors
Source: Bioengineering (Basel). 2023 Nov 27;10(12):1361. doi: 10.3390/bioengineering10121361 (PMC10741009; doi:10.3390/bioengineering10121361)
Supplement: Supplementary file 1 [file bioengineering-10-01361-s001.zip › bioengineering-2659794-supplementary.pdf]

Supporting Information

Synthesis, Characterization, DFT, and In Silico Investigation of Two Newly Synthesized β-Diketone Derivatives as Potent COX-2 Inhibitors

Malahat Musrat Kurbanova [1,*], Abel Mammadali Maharramov [1], Arzu Zabit Sadigova [1], Fidan Zaur Gurbanova [2], Suraj Narayan Mali [3], Rashad Al-Salahi [4], Youness El Bakri [5] and Chin-Hung Lai [6,*]

[1] Organic Chemistry Department, Baku State University, Z. Khalilov 23, Baku 1148, Azerbaijan; amaharramov@bsu.edu.az (A.M.M.); arzu_sadigova@yahoo.com (A.Z.S.)
[2] Department of Pharmacy and Biotechnology, Bioinformatics, University of Bologna, Via Marsala, 49/A, 40126 Bologna, Italy; fidangurbanova95@outlook.com
[3] Department of Pharmaceutical Sciences and Technology, Birla Institute of Technology, Mesra 835215, India; mali.suraj1695@gmail.com
[4] Department of Pharmaceutical Chemistry, College of Pharmacy, King Saud University, Riyadh 11451, Saudi Arabia; ralsalahi@ksu.edu.sa
[5] Department of Theoretical and Applied Chemistry, South Ural State University, Lenin Prospect 76, Chelyabinsk 454080, Russia; yns.elbakri@gmail.com
[6] Department of Medical Applied Chemistry, Chung Shan Medical University, Taichung 40241, Taiwan
* Correspondence: mkurbanova72@mail.ru (M.M.K.); chlai125@csmu.edu.tw (C.-H.L.); Tel.: +886-4-24730022-12224 (C.-H.L.)

Table of Contents:

1. The B3LYP-optimized geometries of **1** and **2**
2. The checkcif file of **1**
3. The checkcif file of **2**
4. The whole amino acid sequences of COX-1 and COX-2 enzymes

The DFT-converged geometry of **1**

F	6.44003300	0.32892500	0.17382700
O	-1.02552600	-2.14395400	-0.21865500
O	-1.57581800	2.55395600	-0.15949900
N	0.14037300	0.45106400	-0.15591100
N	0.99590500	-0.51967000	-0.11333300
H	0.62948800	-1.48187700	-0.13550100
C	-1.73879600	-1.13326900	-0.27226900
C	-1.16043200	0.21263900	-0.23445900
C	-2.02621900	1.43000500	-0.26151000
C	-3.51977400	1.20042300	-0.45368900
H	-3.69724500	1.19022800	-1.53843400
H	-4.03667100	2.07841300	-0.05937300
C	-4.06540700	-0.10420100	0.16043200
C	-3.24146000	-1.27770600	-0.40631600
H	-3.52023600	-2.23023600	0.05216300
H	-3.44583600	-1.37870400	-1.48159100
C	2.38015300	-0.26825200	-0.04113000
C	2.88770100	1.03493700	-0.00614300
H	2.20416300	1.87285900	-0.03547700
C	4.26077800	1.23317100	0.06662000
H	4.68171300	2.23031300	0.09534200
C	5.10253500	0.12972900	0.10303300
C	4.61951300	-1.16881500	0.06924000
H	5.30849000	-2.00312500	0.09940000
C	3.24459800	-1.36663400	-0.00360200
H	2.84453200	-2.37421800	-0.03121400
C	-5.54051900	-0.28159700	-0.23439500
H	-5.66059700	-0.31090200	-1.32164500
H	-6.14788200	0.54371700	0.14861900
H	-5.94606400	-1.21206900	0.17463800
C	-3.95536100	-0.06229800	1.69769100
H	-2.92307200	0.06075100	2.03754600
H	-4.34233300	-0.98584500	2.13812100
H	-4.53580100	0.77179900	2.10188000

The DFT-converged geometry of **2**

F	-2.45513800	-1.97582700	-1.13724500
O	0.94286400	-1.77123000	-0.18327800
O	2.37472000	2.73537500	-0.17013700
N	-0.74373800	0.21526700	-0.08551700
H	-0.57792800	-0.79969100	-0.09752500
N	0.29618900	0.99671400	-0.14424800
C	-2.03205900	0.77947200	-0.00443700

C	-3.17583200	-0.04400600	0.04131100
C	-4.44058500	0.53965100	0.12395300
H	-5.31196100	-0.10011500	0.15848700
C	-4.58634300	1.92142600	0.16132600
H	-5.57470600	2.35940200	0.22570600
C	-3.45232900	2.73078900	0.11523800
H	-3.55187400	3.80968600	0.14389900
C	-2.18536100	2.17033400	0.03327400
H	-1.29944200	2.78912900	-0.00194800
C	1.51898500	0.51488900	-0.22897100
C	1.83450800	-0.92319300	-0.25838100
C	3.28133700	-1.34622300	-0.41025000
H	3.44966700	-1.48229800	-1.48794500
H	3.38279300	-2.33426200	0.04625400
C	4.31572500	-0.34620600	0.14451300
C	4.02332100	1.03535600	-0.47517700
H	4.70300300	1.80064200	-0.09324600
H	4.18372400	0.98432100	-1.56149200
C	2.60408300	1.54728300	-0.27125500
C	-3.06121500	-1.54479100	-0.00066900
C	5.72805400	-0.80091600	-0.25754100
H	5.83309100	-0.85833100	-1.34520200
H	5.95313300	-1.78898300	0.15525700
H	6.48283800	-0.10316300	0.11679600
C	4.22490300	-0.27617200	1.68198900
H	4.43346800	-1.25369100	2.12625100
H	3.23616600	0.04015200	2.02685100
H	4.95416100	0.43595900	2.07823400
F	-4.25796200	-2.15788200	0.06030100
F	-2.31880100	-2.02650500	1.02980600

checkCIF/PLATON report

You have not supplied any structure factors. As a result the full set of tests cannot be run.

THIS REPORT IS FOR GUIDANCE ONLY. IF USED AS PART OF A REVIEW PROCEDURE FOR PUBLICATION, IT SHOULD NOT REPLACE THE EXPERTISE OF AN EXPERIENCED CRYSTALLOGRAPHIC REFEREE.

No syntax errors found. CIF dictionary Interpreting this report

Datablock: A

```
Bond precision:  C-C = 0.0031 A         Wavelength=0.71073

Cell:          a=5.993(2)        b=10.446(4)       c=10.731(4)
               alpha=97.765(8)   beta=102.860(8)   gamma=98.925(8)
Temperature:   296 K

                Calculated                 Reported
Volume          636.9(4)                   637.0(4)
Space group     P -1                       P -1
Hall group      -P 1                       -P 1
Moiety formula  C14 H15 F N2 O2            C14 H15 F N2 O2
Sum formula     C14 H15 F N2 O2            C14 H15 F N2 O2
Mr              262.28                     262.28
Dx,g cm-3       1.368                      1.368
Z               2                          2
Mu (mm-1)       0.102                      0.102
F000            276.0                      276.0
F000'           276.14
h,k,lmax        7,12,13                    7,12,13
Nref            2506                       2454
Tmin,Tmax       0.969,0.978                0.424,0.747
Tmin'           0.967

Correction method= # Reported T Limits: Tmin=0.424 Tmax=0.747
AbsCorr = MULTI-SCAN

Data completeness= 0.979          Theta(max)= 25.997

R(reflections)= 0.0774( 2121)     wR2(reflections)= 0.2627( 2454)

S = 1.041                 Npar= 174
```

The following ALERTS were generated. Each ALERT has the format
test-name_ALERT_alert-type_alert-level.
Click on the hyperlinks for more details of the test.

Alert level C

```
PLAT084_ALERT_3_C High wR2 Value (i.e. > 0.25) ...................       0.26 Report
PLAT112_ALERT_2_C ADDSYM Detects New (Pseudo) Symm. Elem.       B          84 %Fit
```

Alert level G

```
PLAT007_ALERT_5_G Number of Unrefined Donor-H Atoms .............          1 Report
PLAT072_ALERT_2_G SHELXL First  Parameter in WGHT  Unusually Large      0.18 Report
PLAT154_ALERT_1_G The s.u.'s on the Cell Angles are Equal ..(Note)     0.008 Degree
PLAT933_ALERT_2_G Number of OMIT records in Embedded RES .........         9 Note
```

```
   0 ALERT level A = Most likely a serious problem - resolve or explain
   0 ALERT level B = A potentially serious problem, consider carefully
   2 ALERT level C = Check. Ensure it is not caused by an omission or oversight
   4 ALERT level G = General information/check it is not something unexpected

   1 ALERT type 1 CIF construction/syntax error, inconsistent or missing data
   3 ALERT type 2 Indicator that the structure model may be wrong or deficient
   1 ALERT type 3 Indicator that the structure quality may be low
   0 ALERT type 4 Improvement, methodology, query or suggestion
   1 ALERT type 5 Informative message, check
```

Publication of your CIF

A full structural check has been run on your CIF. This includes checks on:

- CIF syntax and construction
- Cell and geometry details
- Space-group symmetry
- Anisotropic displacement parameters

Structure-factor checking is currently being tested on articles submitted to *Acta Crystallographica Section C* and *Acta Crystallographica Section E*. These tests may be carried out with a local version of PLATON or the trial service here.

These full checks give an indication of potential problems with your CIF. Please note that if you intend to submit your CIF for publication in Acta Crystallographica Section C or E or IUCrData, you must make sure that full publication checks are run on the final version of the CIF prior to submission.

If you intend to submit to another section of Acta Crystallographica, Journal of Applied Crystallography or Journal of Synchrotron Radiation, you should make sure that at least basic structural checks are run on the final version of your CIF prior to submission.

To submit your CIF for publication in an IUCr journal click here.

PLATON version of 11/08/2016; check.def file version of 04/08/2016

Datablock A - ellipsoid plot

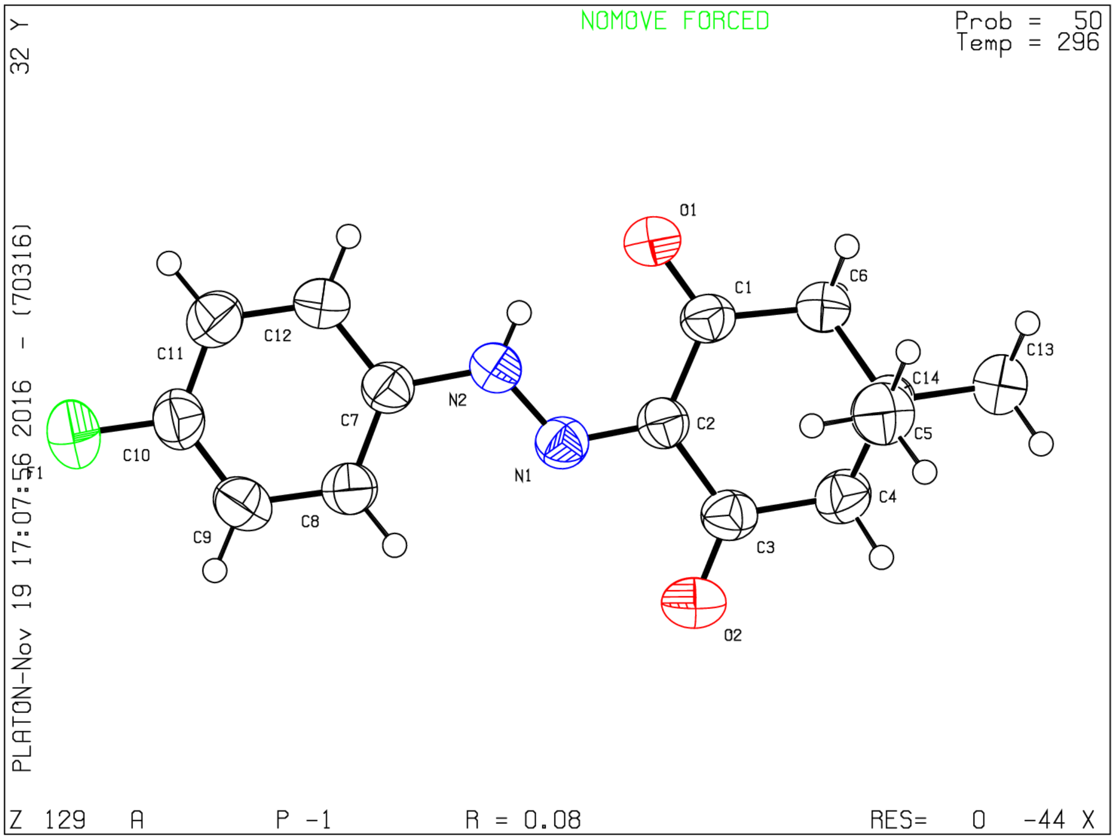

checkCIF/PLATON report

You have not supplied any structure factors. As a result the full set of tests cannot be run.

THIS REPORT IS FOR GUIDANCE ONLY. IF USED AS PART OF A REVIEW PROCEDURE FOR PUBLICATION, IT SHOULD NOT REPLACE THE EXPERTISE OF AN EXPERIENCED CRYSTALLOGRAPHIC REFEREE.

No syntax errors found. CIF dictionary Interpreting this report

Datablock: ma2

```
Bond precision:  C-C = 0.0020 A          Wavelength=0.71073

Cell:           a=15.5610(12)    b=6.1069(5)          c=15.6267(12)
                alpha=90         beta=97.3588(13)     gamma=90
Temperature:    564 K

                Calculated                 Reported
Volume          1472.8(2)                  1472.8(2)
Space group     P 21/n                     P 21/n
Hall group      -P 2yn                     -P 2yn
Moiety formula  C15 H15 F3 N2 O2           C15 H15 F3 N2 O2
Sum formula     C15 H15 F3 N2 O2           C15 H15 F3 N2 O2
Mr              312.29                     312.29
Dx,g cm-3       1.408                      1.408
Z               4                          4
Mu (mm-1)       0.120                      0.120
F000            648.0                      648.0
F000'           648.43
h,k,lmax        20,8,20                    20,8,20
Nref            3543                       3528
Tmin,Tmax       0.969,0.982                0.635,0.746
Tmin'           0.927

Correction method= # Reported T Limits: Tmin=0.635 Tmax=0.746
AbsCorr = MULTI-SCAN

Data completeness= 0.996          Theta(max)= 28.000

R(reflections)= 0.0442( 2051)     wR2(reflections)= 0.1083( 3528)

S = 1.085                  Npar= 233
```

The following ALERTS were generated. Each ALERT has the format
test-name_ALERT_alert-type_alert-level.
Click on the hyperlinks for more details of the test.

Alert level C

```
ABSTY02_ALERT_1_C  An _exptl_absorpt_correction_type has been given without
           a literature citation. This should be contained in the
           _exptl_absorpt_process_details field.
           Absorption correction given as multi-scan
PLAT230_ALERT_2_C Hirshfeld Test Diff for    C2     --  C13     ..        6.3 s.u.
PLAT230_ALERT_2_C Hirshfeld Test Diff for    C10    --  C15     ..        6.1 s.u.
PLAT242_ALERT_2_C Low    'MainMol' Ueq as Compared to Neighbors of        C10 Check
```

Alert level G

```
PLAT002_ALERT_2_G Number of Distance or Angle Restraints on AtSite          7 Note
PLAT003_ALERT_2_G Number of Uiso or Uij Restrained non-H Atoms ...          6 Report
PLAT063_ALERT_4_G Crystal Size Likely too Large for Beam Size ....       0.63 mm
PLAT172_ALERT_4_G The CIF-Embedded .res File Contains DFIX Records          1 Report
PLAT186_ALERT_4_G The CIF-Embedded .res File Contains ISOR Records          1 Report
PLAT242_ALERT_2_G Low    'MainMol' Ueq as Compared to Neighbors of        C13 Check
PLAT301_ALERT_3_G Main Residue  Disorder ............ Percentage =         14 Note
PLAT860_ALERT_3_G Number of Least-Squares Restraints ............          42 Note
```

```
   0 ALERT level A = Most likely a serious problem - resolve or explain
   0 ALERT level B = A potentially serious problem, consider carefully
   4 ALERT level C = Check. Ensure it is not caused by an omission or oversight
   8 ALERT level G = General information/check it is not something unexpected

   1 ALERT type 1 CIF construction/syntax error, inconsistent or missing data
   6 ALERT type 2 Indicator that the structure model may be wrong or deficient
   2 ALERT type 3 Indicator that the structure quality may be low
   3 ALERT type 4 Improvement, methodology, query or suggestion
   0 ALERT type 5 Informative message, check
```

Publication of your CIF

A full structural check has been run on your CIF. This includes checks on:

- CIF syntax and construction
- Cell and geometry details
- Space-group symmetry
- Anisotropic displacement parameters

Structure-factor checking is currently being tested on articles submitted to *Acta Crystallographica Section C* and *Acta Crystallographica Section E*. These tests may be carried out with a local version of PLATON or the trial service here.

These full checks give an indication of potential problems with your CIF. Please note that if you intend to submit your CIF for publication in Acta Crystallographica Section C or E or IUCrData, you must make sure that full publication checks are run on the final version of the CIF prior to submission.

If you intend to submit to another section of Acta Crystallographica, Journal of Applied Crystallography or Journal of Synchrotron Radiation, you should make sure that at least basic structural checks are run on the final version of your CIF prior to submission.

To submit your CIF for publication in an IUCr journal click here.

PLATON version of 11/08/2016; check.def file version of 04/08/2016

Datablock ma2 - ellipsoid plot

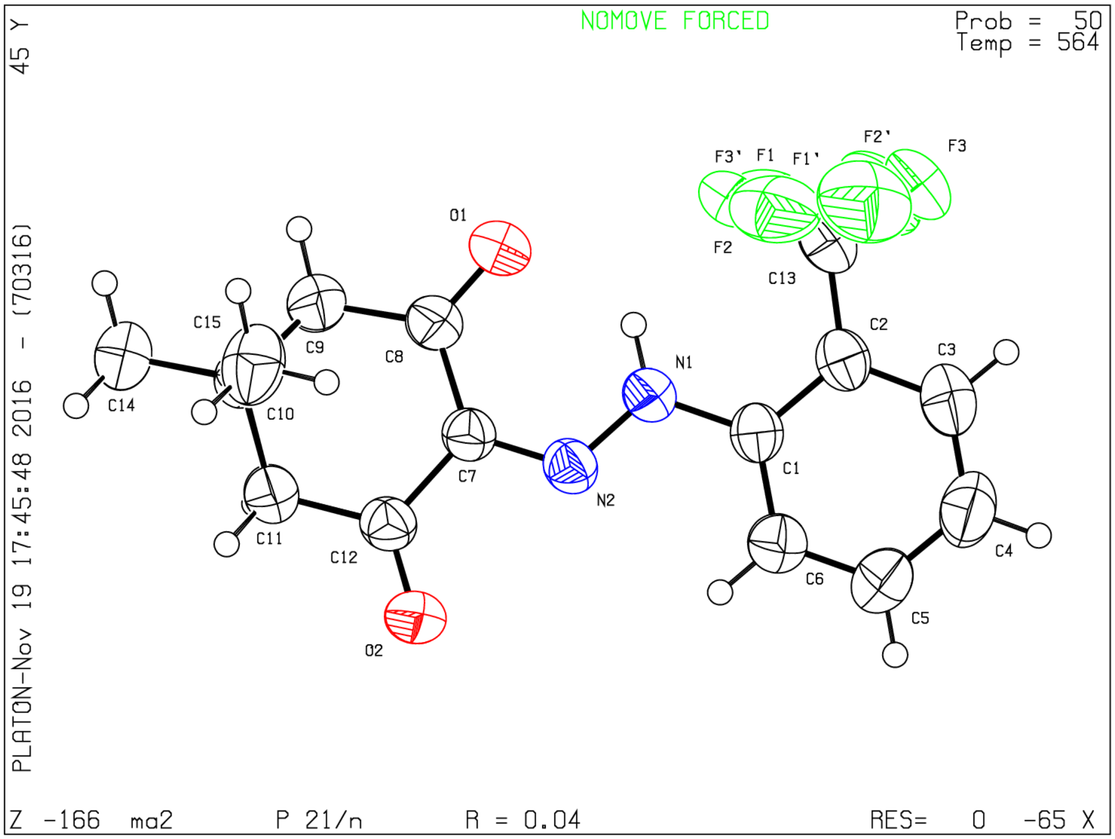

COX-1

MSRSLLLRFLLFLLLLPPLPVLLADPGAPTPVNPCCYYPCQHQGICVRFGLDRYQCDCTRTGYSGP
NCTIPGLWTWLRNSLRPSPSFTHFLLTHGRWFWEFVNATFIREMLMRLVLTVRSNLIPSPPTYNS
AHDYISWESFSNVSYYTRILPSVPKDCPTPMGTKGKKQLPDAQLLARRFLLRRKFIPDPQGTNLM
FAFFAQHFTHQFFKTSGKMGPGFTKALGHGVDLGHIYGDNLERQYQLRLFKDGKLKYQVLDGE
MYPPSVEEAPVLMHYPRGIPPQSQMAVGQEVFGLLPGLMLYATLWLREHNRVCDLLKAEHPT
WGDEQLFQTTRLILIGETIKIVIEEYVQQLSGYFLQLKFDPELLFGVQFQYRNRIAMEFNHLYHWH
PLMPDSFKVGSQEYSYEQFLFNTSMLVDYGVEALVDAFSRQIAGRIGGGRNMDHHILHVAVDVI
RESREMRLQPFNEYRKRFGMKPYTSFQELVGEKEMAAELEELYGDIDALEFYPGLLLEKCHPNSIF
GESMIEIGAPFSLKGLLGNPICSPEYWKPSTFGGEVGFNIVKTATLKKLVCLNTKTCPYVSFRVPDA
SQDDGPAVERPSTEL

COX-2

MLARALLLCAVLALSHTANPCCSHPCQNRGVCMSVGFDQYKCDCTRTGFYGENCSTPEFLTRIKL
FLKPTPNTVHYILTHFKGFWNVVNNIPFLRNAIMSYVLTSRSHLIDSPPTYNADYGYKSWEAFSNL
SYYTRALPPVPDDCPTPLGVKGKKQLPDSNEIVEKLLLRRKFIPDPQGSNMMFAFFAQHFTHQFF
KTDHKRGPAFTNGLGHGVDLNHIYGETLARQRKLRLFKDGKMKYQIIDGEMYPPTVKDTQAEM
IYPPQVPEHLRFAVGQEVFGLVPGLMMYATIWLREHNRVCDVLKQEHPEWGDEQLFQTSRLILI
GETIKIVIEDYVQHLSGYHFKLKFDPELLFNKQFQYQNRIAAEFNTLYHWHPLLPDTFQIHDQKYN
YQQFIYNNSILLEHGITQFVESFTRQIAGRVAGGRNVPPAVQKVSQASIDQSRQMKYQSFNEYRK
RFMLKPYESFEELTGEKEMSAELEALYGDIDAVELYPALLVEKPRPDAIFGETMVEVGAPFSLKGLM
GNVICSPAYWKPSTFGGEVGFQIINTASIQSLICNNVKGCPFTSFSVPDPELIKTVTINASSSRSGLD
DINPTVLLKERSTEL
